# Biomethylation and Volatilization of Arsenic by Model Protozoan *Tetrahymena pyriformis* under Different Phosphate Regimes

**DOI:** 10.3390/ijerph14020188

**Published:** 2017-02-14

**Authors:** Xixiang Yin, Lihong Wang, Zhanchao Zhang, Guolan Fan, Jianjun Liu, Kaizhen Sun, Guo-Xin Sun

**Affiliations:** 1 Jinan Research Academy of Environmental Sciences, Jinan 250014, China; xxyincas@gmail.com (X.Y.); zhanchaozhang123@gmail.com (Z.Z.); glfan410@gmail.com (G.F.); jjlou418@gmail.com (J.L.); kzsun@mail.sdu.edu.cn (K.S.); 2 Key Laboratory of Urban Environment and Health, Institute of Urban Environment, Chinese Academy of Sciences, Xiamen 361021, China; 3 Shandong Analysis and Test Center, Shandong Academy of Sciences, Jinan 250014, China; wlhkxy@gmail.com; 4 State Key Laboratory of Urban and Regional Ecology, Research Center for Eco-Environmental Sciences, Chinese Academy of Sciences, Beijing 100085, China

**Keywords:** arsenic, accumulation, biotransformation, phosphate, protozoan, *Tetrahymena pyriformis*

## Abstract

*Tetrahymena pyriformis*, a freshwater protozoan, is common in aquatic systems. Arsenic detoxification through biotransformation by *T. pyriformis* is important but poorly understood. Arsenic metabolic pathways (including cellular accumulation, effluxion, biomethylation, and volatilization) of *T. pyriformis* were investigated at various phosphate concentrations. The total intracellular As concentration increased markedly as the external phosphate concentration decreased. The highest concentration was 168.8 mg·kg^−1^ dry weight, after exposure to As(V) for 20 h. Inorganic As was dominant at low phosphate concentrations (3, 6, and 15 mg·L^−1^), but the concentration was much lower at 30 mg·L^−1^ phosphate, and As(V) contributed only ~7% of total cellular As. Methylated As contributed 84% of total As at 30 mg·L^−1^ phosphate, and dimethylarsenate (DMAs(V)) was dominant, contributing up to 48% of total As. Cellular As effluxion was detected, including inorganic As(III), methylarsenate (MAs(V)) and DMAs(V). Volatile As was determined at various phosphate concentrations in the medium. All methylated As concentrations (intracellular, extracellular, and volatilized) had significant linear positive relationships with the initial phosphate concentration. To the best of our knowledge, this is the first study of As biotransformation by protozoa at different phosphate concentrations.

## 1. Introduction

Arsenic is a carcinogen that is widely found in aquatic environments such as rivers and seas. Drinking water contaminated with As is a major health problem that is causing concern in a number of places. Arsenic concentrations in drinking water in many parts of the world, such as parts of Bangladesh, China, India, and Vietnam, are higher than the maximum concentrations of 10 μg·L^−1^ specified by the World Health Organization [1,2,3]. Millions of people in these areas suffer from excessive exposure to As. Arsenic can enter the food chain in crops irrigated with contaminated groundwater [4].

The environmental fate of As and toxicity of As to organisms depend on the speciation of the As present. Inorganic As is mainly present in the pentavalent and trivalent forms, As(V) and As(III), respectively, in aquatic ecosystems. Many studies of the biotransformation of inorganic As by microorganisms and plants have been performed [5]. The reduction of arsenate, i.e., the reduction of As(V) to As(III), within cells is a crucial step in the biotransformation of inorganic As. The reduced product As(III) is involved in various bio-metabolic pathways and can be oxidized to As(V) or transformed into methylated arsenic species. Methylated As species are less toxic than inorganic As, so the generation of the organic As species methylarsenate (MAs(V)), dimethylarsenate (DMAs(V)) and trimethylarsine oxide (TMAs(V)O) as well as trimethylarsine (TMAs) is considered to be an effective pathway through which organisms detoxify As [6]. The biovolatilization of inorganic As is seen as an important metabolic approach used by organisms [7,8]. It has been found in previous studies that some green microalgae and bacteria have strong capacities to volatilize As [6,7,8,9].

Phosphorus is an essential nutrient that is involved in the growth and development of cells, accounting for about 2%–4% of the dry weight of cells. Increasing P levels in the soil due of human actions, such as fertilizers and animal feeds, elevate the P runoff to aquatic ecosystems and cause P eutrophication [10]. Phosphorus concentration in aquatic environments could reach μM levels. Phosphate and arsenate are chemically similar, so they are absorbed competitively by microorganisms. The P concentration in the environment of a microorganism can affect the accumulation and transformation of As by the microorganism and the toxicity of As to the microorganism [11,12]. There have been many reports of As(V) being taken up by eukaryotic plants through the phosphate pathway, and the processes involved are relatively well understood. The mechanism through which inorganic As(V) is taken up by aquatic microorganisms in environments containing different P concentrations has also been studied [13]. The results of a previous study indicated that As(V) may activate phosphate sensors and disturb the phosphate signal pathway, which could cause an organism to display phosphate starvation responses through repressing genes [14].

*Tetrahymena pyriformis* is a freshwater protozoan that is found in various aquatic systems in which P and As are both commonly present. The sensitivity of *T. pyriformis* to environmental contamination has led to it being used widely as a model organism to monitor aquatic toxicity and assess environmental risks [15]. It has been found that *T. pyriformis* can survive in the presence of 40 μM arsenate, which is far higher than realistic environmental concentrations of arsenate [16]. In some lakes such as Mono Lake and Searles Lake in California, the concentrations of As are up to 200 μM and 3000 μM, respectively [17]. The aim of the study described here was to investigate the metabolism of As in *T. pyriformis* cells under different phosphate regimes because of the importance of P to cell growth, the pervasiveness of As in the environment, and the crucial aquatic ecological niches protozoa occupy. Competition between phosphate and As(V) accumulation and transport in bacteria and plants has been demonstrated in numerous studies, but the effect of phosphate on the biotransformation of As(V) by protozoa has been little studied.

## 2. Materials and Methods

### 2.1. Culturing T. pyriformis

A culture of *T. pyriformis* was acquired from the Institute of Urban Environment, Chinese Academic of Sciences. The culture was kept at 30 °C, without shaking, in a modified Neff medium consisting of 0.25% proteose peptone (BD, Franklin Lakes, NJ, USA), 0.5% glucose (Sinopharm, Beijing, China), 0.25% Difco yeast extract (Thermo Fisher Scientific, Waltham, MA, USA), and 3.33 μM FeCl_3_.

### 2.2. Growth of T. pyriformis Exposed to As at Different Phosphate Concentrations

Cells were harvested in the early exponential phase (21 h post-inoculation), and washed with sterile Milli-Q water. Pellets of the cells were inoculated into 250 mL flasks containing Neff medium with phosphate added. Na_3_AsO_4_ was added to give a concentration of 40 μM, and the phosphate concentrations in the different flasks were 3, 6, 15, and 30 mg·L^−1^. This allowed the effects of As toxicity on the cells in media containing different phosphate concentrations to be investigated. Growth was determined by measuring the optical density at 600 nm (OD_600_) after the cells had been exposed to As(V) for 20 h. Three replicate experiments were performed at each phosphate concentration.

### 2.3. Total As Concentrations and Speciation in the Cells and Media

The cultures were incubated for 20 h, then each culture was centrifuged at 4000× *g* and 0 °C for 5 min and samples of the cells and supernatant were collected. The cells were washed with sterile Milli-Q water, then stored at −80 °C.

The total cellular As and P concentrations in each cell sample were determined. Approximately 0.01 g dry weight (DW) of a cell sample was weighed and transferred into a 50 mL polypropylene digestion tube, then 2 mL of concentrated nitric acid was added and the mixture left overnight. Quality control was achieved by analyzing three standard reference material GBW10010 (rice flour) samples and three reagent blanks in the same way as the samples. The tubes containing the partly digested mixtures were heated in a microwave-accelerated reaction system (CEM Microwave Technology, Buckingham, UK) using a three-stage temperature ramping program that has been described previously [18]. The samples were then cooled, and each digest was diluted to 50 mL with Milli-Q water. The solutions were then stored at −20 °C until they were analyzed [16]. The total As and total P concentrations in the digests were determined using an inductively-coupled plasma mass spectrometer (Agilent 7500 ICP-MS; Agilent Technologies, Santa Clara, CA, USA) [16]. The total As in each medium (i.e., supernatant) sample was also determined. A 5 mL aliquot of the supernatant was passed through a 0.45-μm filter, acidified by adding 50 μL concentrated nitric acid, then stored at −4 °C until analysis.

The speciation of the As in each cell sample was investigated using the following procedure. Approximately 0.01 g DW of a cell sample was weighed and transferred to a 50-mL polypropylene digestion tube, then 8 mL of 1% nitric acid was added and the mixture was left for 10 h. A 5-mL aliquot of the supernatant was passed through a 0.45-μm filter, then the extract was analyzed by high performance liquid chromatography coupled with inductively-coupled plasma mass spectrometer (ICP-MS, Agilent Technologies). The As species were separated using a PRP-X100 anion exchange column (Hamilton, Reno, NV, USA). The aqueous mobile phase contained 6.66 mmol·L^−1^ NH_4_NO_3_ and 6.66 mmol·L^−1^ (NH_4_)_2_HPO_4_, and the pH was adjusted to 6.2 by adding ammonia solution.

The total As and total P limits of detection (LOD) were 0.013 and 0.009 μg·L^−1^, respectively. The As and P recoveries for the standard reference materials were 107% and 103%, respectively. The limits of detection for the different arsenic species (As(III), As(V), DMAs(V), and MMAs(V)) were all 70 ng·As·L^−1^.

### 2.4. Chemotrapping of Volatile Arsenic

**Figure 1:**
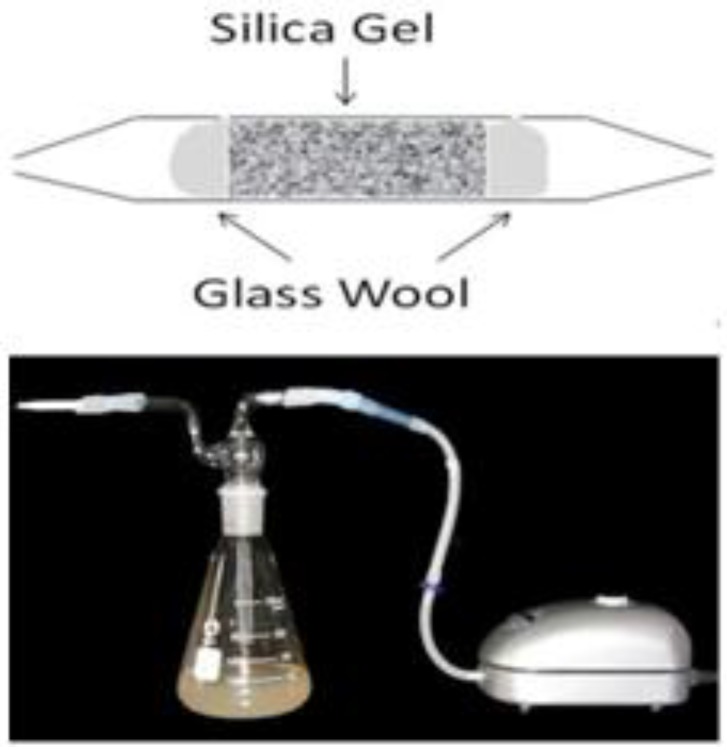
The chemo-trapping for volatile arsenicals. Silica gel (0.4-mm diameter) was steeped with 10% AgNO_3_ solution (*w*/*v*) overnight, and dried at 70 °C; oxygen was supplied with an adjustable air pump for the cell growth.

The volatile As species produced by *T. pyriformis* were trapped in our previous study [16]. The trap device used in the study described here is shown in Figure 1. The volatile As concentration in a trapped sample was determined using the method we have described previously [16].

### 2.5. Scanning Electron Microscopy (SEM) Analysis

The effects of different As species on the morphological characteristics of *T. pyriformis* cells were identified by performing scanning electron microscopy on cells cultured in 40 μM Na_3_AsO_4_ in media containing P concentrations at 3 or 30 mg·L^−1^. As in the experiment described above, the cells were incubated for 21 h, then arsenate was added and the cells were cultured at 25 °C for 20 h. Each culture was then centrifuged and the supernatant discarded. The cells were fixed with 2.5% glutaric dialdehyde overnight, then washed with 0.1 M phosphate buffer and dehydrated using ethanol. Each cell sample was then dried and coated with metal using a Polaron SC7620 sputter coater (Quorum Technologies, Ashford, UK), then observed using a Hitachi S-2000N scanning electron microscope (SEM) (Hitachi High-Technologies, Tokyo, Japan).

## 3. Results and Discussion

### 3.1. Toxic Effects of As in T. pyriformis at Different Phosphate Concentrations

**Figure 2:**
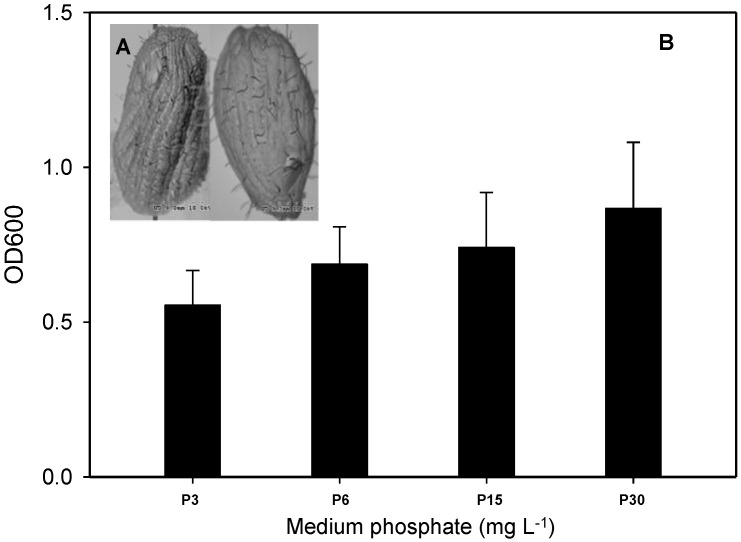
Growth of *Tetrahymena pyriformis* exposed to various P concentrations after 20 h. (**A**) Scanning electron microscopic photographs of *T. pyriformis*. Left, 3 mg·L^−1^ P treatment; Right, 30 mg·L^−1^ P treatment; (**B**) Growth of *T. pyriformis* cell. All data are means ± SE (*n* = 4). OD: optical density.

Similar trends of As transformation by *T. pyriformis* at high (40 μM) and low (2 μM) As concentrations were observed in our previous study [16], and the flux of volatilized As at high concentration (9.3 ng·mg^−1^·day^−1^) was much higher than that at low concentration (1.3 ng·mg^−1^·day^−1^). A high-level concentration (40 μM) was therefore chosen for further investigation in this study. The morphologies of *T. pyriformis* cells that had been exposed to 40 μM arsenate and two different phosphate concentrations were investigated by scanning electron microscopy to allow us to gain an understanding of the toxic effects caused by As(V). The microscopy images are shown in Figure 2A. No significant differences were observed in the morphological characteristics of the cells exposed at the two different phosphate concentrations (3 and 30 mg·L^−1^), and no obvious differences were found in the cell membranes, pores, and flagella. We also investigated the growth of *T. pyriformis* incubated for 20 h in 40 μM arsenate and different phosphate concentrations. Cell growth was retarded by 14.6%–30.3% at the lower phosphate concentrations (3, 6, and 15 mg·L^−1^) compared with the cell growth rate at 30 mg·L^−1^ phosphate (Figure 2B). It has been found in previous studies that As(V) can inhibit the growth of some bacterium and plant cells at low phosphate concentrations [12,19,20]. It is possible that excessive intracellular As(V) decreases the effectiveness of the cellular energy source, causing cell death. Competitive absorption between As and P means that higher external phosphate concentrations can effectively decrease As(V) toxicity by inhibiting intracellular accumulation of As(V), meaning that the cells can grow better than at lower phosphate concentrations (Figure 2B).

### 3.2. Effects of Phosphate on As Accumulation and Biotransformation in T. pyriformis

**Figure 3:**
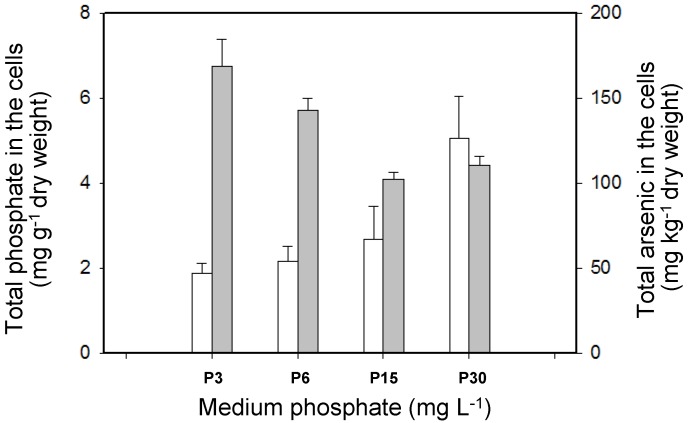
Total concentrations of As and P, when exposed to 40 μM As(V) under different P levels for 20 h incubation. White columns: phosphate levels; gray columns: arsenic levels. All data are the means ± SE (*n* = 4).

The total As concentrations in the cells increased markedly as the external phosphate concentration decreased, reaching 168.84 mg·kg^−1^ DW at the lowest phosphate concentration, 3 mg·L^−1^ (Figure 3). It has been reported that As(V) is absorbed via P transporters, which would explain the competitive effect between As(V) and P uptake [12,20]. The cellular P concentrations tended to increase as the phosphate concentration in the medium increased. Similar results have been reported for some photosynthetic algae and higher plants [11,21].

**Figure 4:**
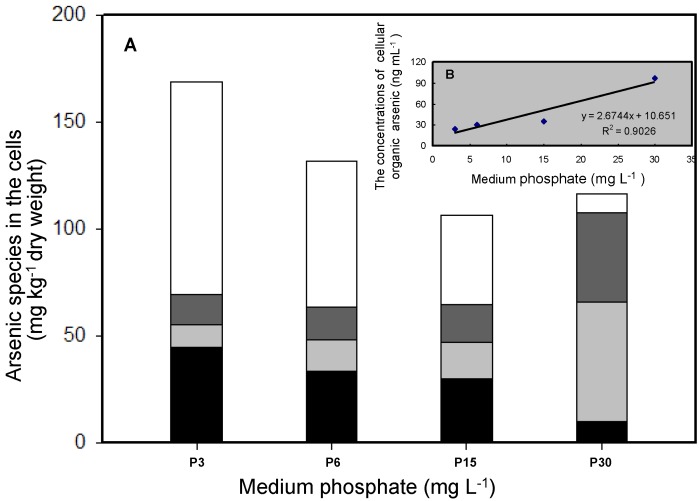
Distribution of As species in the cell after 20 h As(V) exposure under various P levels. (**A**) Concentrations of four arsenic species. Black: As(III); dark gray: dimethylarsenate (DMAs(V)); gray: monomethylarsenate (MMAs(V)); white: As(V); (**B**) The relationship between intracellular organic As concentrations and the initial P levels. All data are the means ± SE (*n* = 4).

The concentrations of different As species in the cells were also determined (Figure 4). Inorganic As (As(III) and As(V)) and two organic arsenic species (MMAs(V) and DMAs(V)) were detected in the cells that had been exposed to different phosphate concentrations. The methylation (and therefore detoxification) of accumulated As by *T. pyriformis* may have a number of steps. As(V), which is the predominant As species in aerobic environments, may first be absorbed by the cells, then reduced to As(III), which is then methylated. At phosphate concentrations of 3, 6, and 15 mg·L^−1^, inorganic As species were the dominant forms within the cells, accounting for 67%–85% of the total As concentrations. As(V) accounted for 39%–59% of the As concentrations. The organic As species MMAs(V) and DMAs(V) accounted for 8%–17% and 6%–16%, respectively, of the total As concentrations in the cells. The percentage of intracellular As(V) concentrations was around 7% of the total As concentrations at the high phosphate concentration of 30 mg·L^−1^, much lower than at the relatively low phosphate concentrations (3, 6, and 15 mg·L^−1^). However, the methylated As concentrations in the cells treated at the high phosphate concentration accounted for 84% of the total As concentrations, and DMAs(V) accounted for 48% of the total As concentrations (Figure 4). There are two crucial As detoxification pathways in aquatic organisms, the reduction of As(V) and the methylation of the As(III) produced [22,23,24]. The results indicated that more cellular arsenate was transformed into arsenite and then methylated to give less toxic organic pentavalent arsenic species at the high phosphate concentration than at the lower phosphate concentrations. The intracellular organic As (MMAs(V) and DMAs(V)) concentrations positively correlated with the initial phosphate level (Figure 3 and Figure 4B). This could be explained by more inorganic As being methylated in the cells in the presence of high phosphate concentrations than in the presence of low phosphate concentrations. An adequate cellular P concentration therefore contributed to the formation of methylated As, and low cellular P concentrations may have decreased cell vitality, impeding the biomethylation process. Similar results have been found for the freshwater alga *Chlorella* sp. and the higher plant *Oryza sativa* [12,25].

**Figure 5:**
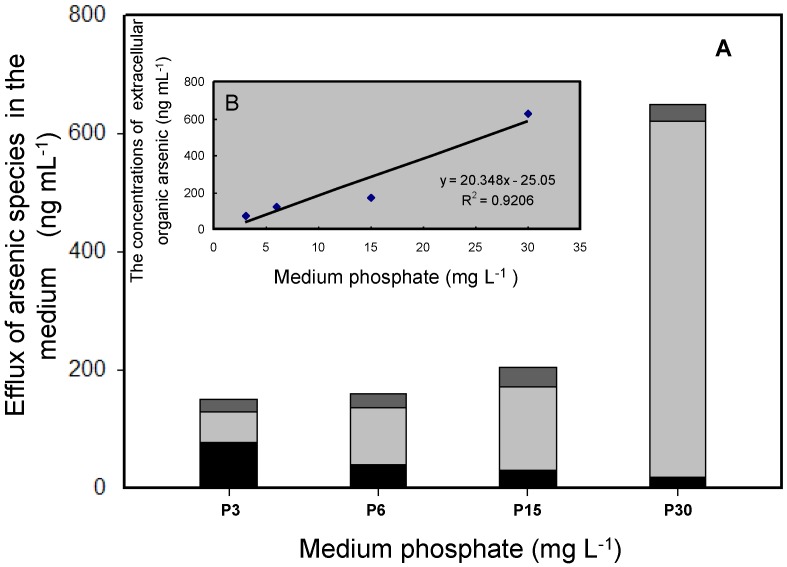
Efflux of As speciation in the medium after cultures were grown for 20 h by *T. pyriformis*. (**A**) Concentrations of As species. Black: As(III); gray: DMAs(V); dark gray: MMAs(V); (**B**) The relationship between extracellular organic As concentrations and the initial P levels. All data are the means ± SE (*n* = 4).

The effluxion of cellular As has been found to be an important detoxification pathway, in addition to reduction and methylation, in many organisms [26]. In our study, methylated As was strongly effluxed by *T. pyriformis* into the culture media at different phosphate concentrations (Figure 5A). We found inorganic As(III) and the two organic arsenic species MMAs(V) and DMAs(V) in the culture media. In the phosphate concentration range 6–30 mg·L^−1^ (i.e., not at the lowest phosphate concentration, 3 mg·L^−1^), the predominant form of As found in the medium was DMAs(V), and As(III) and MMAs(V) accounted for 3%–24% and 4%–15%, respectively, of the total As concentrations. The DMAs(V) concentrations in the media after the cells had been cultured for 20 h clearly increased as the phosphate concentration increased (Figure 5A). There was a significant positive linear correlation between the extracellular organic As concentration and the initial phosphate concentration (Figure 5B). A ready supply of phosphate may accelerate the As(V) reduction and methylation processes and result in the effluxion of methylated As accumulated in the cells. Little As(III) effluxion occurred. Other organisms, such as some eukaryotic algae and bacteria, behave quite differently from *T. pyriformis*, and mainly efflux inorganic As [27,28]. The release of intracellular organic As allows *T. pyriformis* to self-detoxify, and also causes relatively little environmental pressure because DMAs(V) is less toxic than inorganic As species.

### 3.3. Volatilization of As by T. pyriformis at Different Phosphate Concentrations

**Figure 6:**
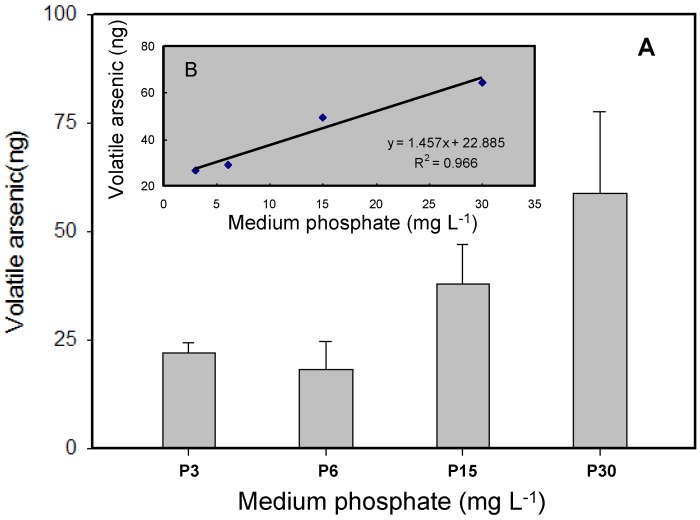
Volatile As level produced by *T. pyriformis.* (**A**) Exposure to 40 μM As(V) for 20 h under different P concentrations; (**B**) The relationship between volatile As and the initial P levels. All data are mean ± SE (*n* = 4).

Volatile As generated by microorganisms has been found to play a crucial role in the biogeochemical cycle of As [23]. It has been found that some bacteria and photosynthetic green alga can volatilize arsenicals by methylating inorganic As and that this acts as a biodetoxification pathway [6,29]. Previous studies have mainly been focused on the production of volatile As by organisms at different As concentrations and after different exposure periods [15,30]. In our study, the potential for As to be biovolatilized by *T. pyriformis* was investigated at different phosphate concentrations, and the results are shown in Figure 6A. When *T. pyriformis* was incubated with 40 μM As(V) for 20 h, the production of volatile As increased as the initial phosphate concentration in the medium increased. At phosphate concentrations of 3 and 6 mg·L^−1^, 26.69 and 29.24 ng, respectively, of volatile As were produced. Much more volatile As was produced at higher external phosphate concentrations, with 49.58 and 64.71 ng of volatile As being found at phosphate concentrations of 15 and 30 mg·L^−1^, respectively. The results indicated that a good supply of phosphate may have increased the methylated As concentrations in the cells, accelerating the generation of volatile As (Figure 4 and Figure 6A). This may be explained by a sufficient supply of phosphate decreasing the cytotoxicity of inorganic As, allowing normal cellular metabolic viability to be maintained and facilitating the production of gaseous As by the cells. It has been found in some studies that a high intracellular methylated As concentration is a key factor in the production of volatile arsenicals [16]. Taking into consideration the importance of the biovolatilization of As in microorganism detoxification and the removal of As from environmental media, *T. pyriformis* seems to be a potential candidate for use in bioremediation of As-polluted aquatic environments.

## 4. Conclusions

*T. pyriformis* had a strong capacity to methylate As and excrete the products to the external environment. The phosphate concentration played a critical role in the metabolism of As by *T. pyriformis*. Arsenic methylation and volatilization by protozoan may be influenced in the presence of phosphate.
